# Plasmacytoid Dendritic Cell, Slan^+^-Monocyte and Natural Killer Cell Counts Function as Blood Cell-Based Biomarkers for Predicting Responses to Immune Checkpoint Inhibitor Monotherapy in Non-Small Cell Lung Cancer Patients

**DOI:** 10.3390/cancers15215285

**Published:** 2023-11-03

**Authors:** Francesca Pettinella, Chiara Lattanzi, Marta Donini, Elena Caveggion, Olivia Marini, Giulia Iannoto, Sara Costa, Elena Zenaro, Tiago Moderno Fortunato, Sara Gasperini, Matteo Giani, Lorenzo Belluomini, Marco Sposito, Jessica Insolda, Ilaria Mariangela Scaglione, Michele Milella, Annalisa Adamo, Ornella Poffe, Vincenzo Bronte, Stefano Dusi, Marco A. Cassatella, Stefano Ugel, Sara Pilotto, Patrizia Scapini

**Affiliations:** 1 General Pathology Section, Department of Medicine, University of Verona, 37134 Verona, Italymarta.donini@univr.it (M.D.); sara.costa@univr.it (S.C.); elena.zenaro@univr.it (E.Z.); fortunato.tiago@gmail.com (T.M.F.); sara.gasperini@univr.it (S.G.); matteo.giani@univr.it (M.G.);; 2 Section of Innovation Biomedicine—Oncology Area, Department of Engineering for Innovation Medicine (DIMI), University of Verona and University and Hospital Trust (AOUI) of Verona, 37134 Verona, Italymarcosposito91@gmail.com (M.S.); ilariascaglione@tiscali.it (I.M.S.); michele.milella@univr.it (M.M.); sara.pilotto@univr.it (S.P.); 3 Immunology Section, Department of Medicine, University and Hospital Trust (AOUI) of Verona, 37134 Verona, Italy; annalisa.adamo@univr.it (A.A.);; 4 Veneto Institute of Oncology—Istituto di Ricovero e Cura a Carattere Scientifico (IOV-IRCCS), 35128 Padova, Italy

**Keywords:** immune checkpoint inhibitors, PD-1/PD-L1, innate immune cells, blood biomarkers, non-small cell lung cancer

## Abstract

**Simple Summary:**

The advent of therapy with immune checkpoint inhibitors (ICIs) has determined a significant survival benefit in patients with non-small cell lung cancer (NSCLC). However, 50–80% of patients do not respond to ICI monotherapy. Therefore, the identification of biomarkers for predicting NSCLC patient response to ICI monotherapy may help to select those who most likely obtain clinical/radiological benefits, in turn reducing the economic impact of health costs. In this study, we identified plasmacytoid dendritic cell, slan^+^-monocyte and natural killer cell counts as encouraging predictive biomarkers for ICI monotherapy in NSCLC patients.

**Abstract:**

The advent of immune checkpoint inhibitors (ICIs), for instance, programmed cell death 1 (PD-1)/PD-1 ligand 1 (PD-L1) blockers, has greatly improved the outcome of patients affected by non-small cell lung cancer (NSCLC). However, most NSCLC patients either do not respond to ICI monotherapy or develop resistance to it after an initial response. Therefore, the identification of biomarkers for predicting the response of patients to ICI monotherapy represents an urgent issue. Great efforts are currently dedicated toward identifying blood-based biomarkers to predict responses to ICI monotherapy. In this study, more commonly utilized blood-based biomarkers, such as the neutrophil-to-lymphocyte ratio (NLR) and the lung immune prognostic index (LIPI) score, as well as the frequency/number and activation status of various types of circulating innate immune cell populations, were evaluated in NSCLC patients at baseline before therapy initiation. The data indicated that, among all the parameters tested, low plasmacytoid dendritic cell (pDC), slan^+^-monocyte and natural killer cell counts, as well as a high LIPI score and elevated PD-L1 expression levels on type 1 conventional DCs (cDC1s), were independently correlated with a negative response to ICI therapy in NSCLC patients. The results from this study suggest that the evaluation of innate immune cell numbers and phenotypes may provide novel and promising predictive biomarkers for ICI monotherapy in NSCLC patients.

## 1. Introduction

Lung cancer is the second most common cancer and the leading cause of cancer-related mortality for men and women (with an estimated 1.8 million deaths comprising 18% of global cancer deaths) [1,2]. The most common histological subtype is non-small cell lung cancer (NSCLC) [3,4]**,** which is recognized as a heterogeneous set of diseases that can benefit from innovative approaches, such as targeted therapies and immunotherapy [5,6,7]. The advent of the immune checkpoint inhibitors (ICIs), including the anti-programmed cell death 1 (PD-1) antibodies, such as nivolumab, pembrolizumab and cemiplimab, and the anti-PD-L1 antibodies, such as atezolizumab or durvalumab, has revolutionized the field of oncology [8,9,10,11]. In fact, ICI monotherapy has outperformed docetaxel in the survival and prognosis of advanced NSCLC patients without driver oncogene mutations**,** and thus has been approved by the Food and Drug Administration (FDA) as a second-line treatment for advanced NSCLC [12,13,14]. In addition, ICI monotherapy demonstrated a survival benefit in the first-line setting, especially in patients with at least 50% of the tumor cells expressing PD-L1, leading to a treatment paradigm shift in clinical practice [11,13,15]. Despite these encouraging results, several patients receiving ICI monotherapy (50–80%) did not respond to the treatment despite a high PD-L1 expression or develop a resistance over time to these therapies [12,16,17]. The mechanisms of the primary and acquired resistance to anti-PD-1 and anti-PD-L1 treatment have been mostly ascribed to changes in the cell composition of the tumor microenvironment (TME)**,** leading to tumor-mediated immunosuppression and immune evasion [16,18,19], which may strongly compromise the clinical efficacy and resistance to the treatment. 

Based on these premises, the identification of baseline characteristics and appropriate biomarkers that could stratify patients into potential responders and non-responders prior to ICI monotherapy treatment is of great clinical significance [20]. In clinical practice, the only currently approved tissue biomarker consists of the evaluation of the PD-L1 tumor proportion score (TPS), which is assessed using immunohistochemistry (patients with a ≥50% PD-L1 expression are generally candidates for ICI monotherapy). By contrast, the evaluation of the tumor mutation burden (TMB) has not yet achieved practical implications for treatment decisions [21,22,23]. However, several limitations, including the intratumoral heterogeneity and the invasiveness of tumor biopsies that cannot be repeatedly performed to monitor early disease response in most patients, render the TPS and TMB as defective and controversial biomarkers [24,25]. On the other hand, monitoring blood-based biomarkers is non-invasive and can be repetitively performed, providing insights to the patient’s immune status [26,27,28]. In the past few years, the number of studies that have evaluated different blood-based parameters as potential predictive biomarkers for ICI therapy in NSCLC patients has grown exponentially [29,30,31,32]. These blood-based biomarkers include parameters such as the serum levels of soluble systemic immune/inflammatory molecules (i.e., the serum levels of lactate dehydrogenase (LDH), C-reactive protein (CRP) or soluble PD-L1), tumor-cell-related markers (i.e., blood-based tumor burden, circulating tumor cells and tumor DNA) or the dynamic changes in the frequency/number of various circulating immune cell subsets [26,29,30,31,32]. In particular, regarding blood-cell-based biomarkers, several studies have evaluated parameters such as the neutrophil-to-lymphocyte ratio (NLR) or the total cell count/frequency of the different subpopulations of lymphocytes or innate immune cells as potential predictive biomarkers for ICI monotherapy in NSCLC patients [27,29,31]. However, especially for the studies on innate immune cells and in particular myeloid-derived suppressor cells (MDSCs) [33,34,35,36,37,38,39,40] and natural killer (NK) cells [34,36,39,41,42], the results are often contradictory. The potential role of the other myeloid cell subsets, such as the monocyte and dendritic cell subsets, as predictive biomarkers for ICI monotherapy has not been fully investigated yet [34,39]. 

Based on these premises, the aim of this work was to better clarify whether the baseline frequency/number and/or activation status of circulating innate immune cell populations, including the neutrophil, monocyte, dendritic cell (DC) and NK cell subpopulations, could function as predictive biomarkers for a response to ICI monotherapy in NSCLC patients. 

## 2. Materials and Methods

### 2.1. Patients and Study Approval

Patients with advanced non-small cell lung cancer (NSCLC) who received ICIs, such as pembrolizumab, nivolumab, durvalumab and atezolizumab as either a first or further line monotherapy, were enrolled in the oncology unit at the University Hospital of Verona. 

We retrospectively defined progressors (P) as the patients who experienced disease progression within six months from the initial administration of ICIs, following at least six weeks of treatment. Conversely, non-progressors (NP) were the patients who did not experience disease progression during treatment after at least six months of clinical or radiological benefits. The baseline blood samples were obtained before initiating the ICI treatment. Prior to the sample collection, all the patients provided written informed consent for the utilization of their clinical and biological data. The overall survival (OS) and progression-free survival (PFS) were determined from the date of the first ICI dose to the date of death or last follow-up and to the evidence of the progression of the disease, respectively. As shown in Table 1, we summarized the clinical and pathological characteristics of the patient cohort. The median age of the participants was 72.5 years (ranging from 43 to 84 years). Among all the patients, 57% had a PD-L1 expression ≥ 50% and 70% had a history of smoking exposure. Only two out of 30 patients harbored an epidermal growth factor (EGFR) mutation. One of these two patients exhibited an exon 19 deletion of the EGFR and underwent first-line targeted therapy followed by subsequent immunotherapy treatment (third line), while the other patient harbored an exon 20 insertion of the EGFR and underwent first-line immunotherapy, considering his PD-L1 levels and clinical conditions. Both patients were included in the study because the values of the blood-based biomarkers found in these patients were comparable to those observed in the patients in the same P group. Adenocarcinoma represents the most frequent histology (77%). The majority of patients were treated using immunotherapy (pembrolizumab) as single agent in the first line (57%). Twenty sex- and age-matched healthy volunteer donors were also enrolled at the blood bank of our institution at the time of blood donation upon informed consent. 

### 2.2. Flow Cytometry Experiments

Blood samples from the study participants were collected in EDTA—or sodium citrate—treated tubes by venipuncture and processed within 1 h. Before being processed for the flow cytometry experiments, the study participant samples underwent complete blood count and blood chemistry measurements (e.g., LDH). 

The mononuclear cells and granulocytes were separated using density gradient centrifugation of the blood onto the Ficoll-Paque gradient (GE Healthcare, Italy) [43]. The low-density neutrophils (LDNs/PMN-MDSCs) were analyzed within the mononuclear cell fraction, while the normal-density neutrophils (NDNs) were analyzed within the granulocyte fraction using flow cytometry, as previously reported [43]. 

Briefly, for the flow cytometry experiments, 2 × 10^5^ cells were resuspended in 20 μL of PBS (Corning Incorporated, Corning, NY, USA) containing 2% FBS (Sigma-Aldrich, St. Louis, MO, USA) and 2 mM EDTA (Sigma-Aldrich) (from now on termed “staining buffer”). The cells were subsequently incubated for 30 min at 4 °C with 5% human serum (Sigma-Aldrich). The cells were then stained for 30 min at 4 °C using the fluorochrome-conjugated monoclonal/polyclonal antibodies (mAbs/pAbs) listed in Table S1. For the immunophenotypic characterization, the data from 150,000 events were acquired using an eight-color three-laser MACSQuant 10 Analyzer or a fourteen-color three-laser MACSQuant 16 Analyzer (Miltenyi Biotec, Bergisch Gladbach, Germany) while the data analysis was performed using the FlowJo software version 10.7.1 (BD Bioscience, San Jose, CA, USA). The phenotypic analysis was performed on live cells identified as Vybrant-negative (Vybran^®^ DyeCycle™ Violet; Life Technologies, Carlsbad, CA, USA), propidium iodide-negative (Merck Life Science, Darmstadt, Germany), Sytox AAdvanced Dead Cell Stain Kit-negative (Invitrogen, Carlsbad, CA, USA), or Fixable Viability Dye eFluor^®^ 450/eFlour^®^660-negative (ThermoFisher Scientific, Waltham, MA, USA). The delta median fluorescence intensity (ΔMFI) relative to CD62L, CD11b, CD35 and PD-L1 was obtained by subtracting either the MFI of the correspondent isotype control or the cell autofluorescence (revealed as Fluorescence Minus One, FMO, control) from the MFI of the specific antibody.

### 2.3. Statistics

The dataset distribution was evaluated using the Shapiro–Wilk normality test. According to the normality test results, the comparison of the variables was performed using an unpaired two-tailed Mann–Whitney test (for comparison between two groups). 

The optimal cut-off value of the selected continuous variables was calculated by performing a receiver operating characteristic (ROC) curve analysis. The selected blood-based parameters were evaluated via univariate analysis for the prediction of the response to the therapy. The ROC curves with an area under the curve (AUC) > 0.5 and a *p*-value < 0.05 were retained for the cut-off selection. The optimal cut-off value was determined based on a trade-off between the specificity and sensitivity. Each variable was changed in dichotomous covariates, according to the selected cutoff value. The ROC curve analysis was performed using the GraphPad Prism Version 9.5 software (GraphPad Software, Inc., La Jolla, CA, USA). To establish the differences in the PFS according to the selected categorical covariates, a univariate Kaplan–Meier curve was performed. The differences among the curves were evaluated using the Wilcoxon test. In addition, a univariate Cox proportional hazard regression model analysis was performed to estimate the hazard ratio (HR). A *p*-value < 0.05 was defined as statistically significant. A survival analysis and a Cox proportional hazard regression model analysis were performed with the survminer and survival R package (R version 4.3). A *p*-value lower than 0.05 was considered significant and asterisks indicated the significant differences: * = *p*-value < 0.05; ** = *p*-value < 0.01; *** = *p*-value < 0.001; **** = *p*-value < 0.0001. The graphs were elaborated using the GraphPad Prism Version 9.5 software (GraphPad Software, Inc.).

## 3. Results

### 3.1. Patients with Advanced NSCLC Responding to ICI Monotherapy Presented Significantly Lower Baseline LIPI Scores, but Not NLR Values, Than Non-Responding NSCLC Patients

**Figure 1:**
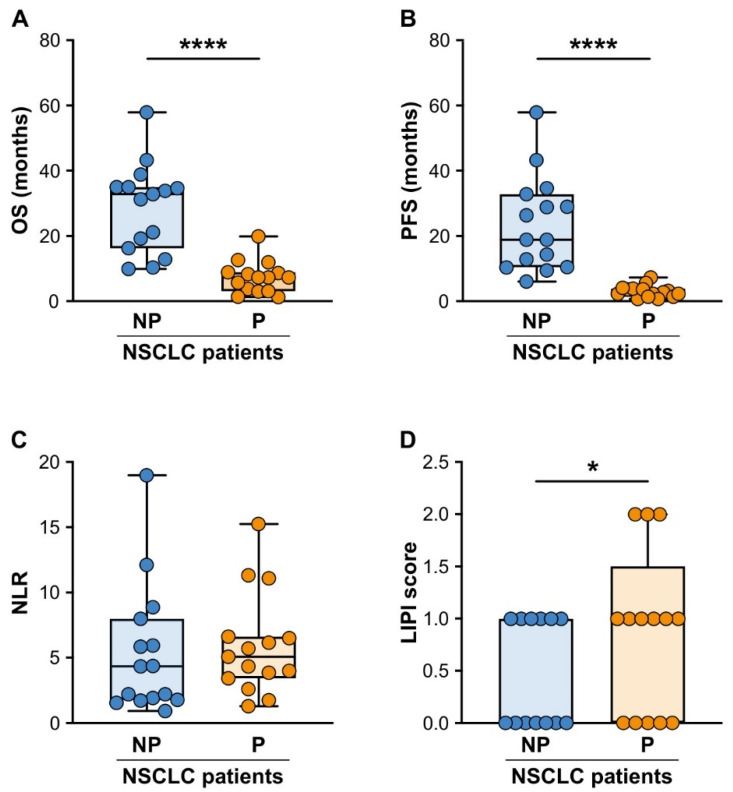
OS, PFS and baseline NLR and LIPI scores of the NP and P advanced NSCLC patients undergoing ICI monotherapy. (**A**,**B**) Box plots showing the overall survival (OS) (**A**) and the progression-free survival (PFS) (**B**), as described in the Materials and Methods, for the NP (*n* = 15) or P (n = 15) NSCLC patients. The median OS and PFS (25th–75th percentile) are reported. (**C**,**D**) Box plots showing the NLR (**C**) and the LIPI scores [which consider the derived NLR and lactate dehydrogenase (LDH) plasma levels] (**D**) in the NP (n = 15–13) or P (n = 15–14) NSCLC patients. The median NLR and LIPI scores (25th–75th percentile) are reported. Each symbol stands for a single NSCLC patient sample. * *p* < 0.05, **** *p* < 0.001; Mann–Whitney test.

In this study, a total of 30 patients with advanced NSCLC treated using ICI monotherapy met the criteria for the final analysis. The clinical and biological characteristics of the patients included in the study are described in the Materials and Methods and summarized in Table 1. At the time of the data cut off, the mean follow-up time was 12.5 months (range, 1.3–58.8 months), with seven patients continuing to receive anti-PD-1/PD-L1 inhibitors. The patients were retrospectively stratified into progressor (P) and non-progressor (NP) groups, according to the criteria defined in the Materials and Methods and as described previously [44]. As expected, the P patients had a significantly lower overall survival (OS) (33 (17.9–35.2) vs. 7.33 (3.47–8.88) months; median OS (25th–75th percentile) for NP patients vs. P patients, respectively; Figure 1A) and progression-free survival (PFS) (19 (11.7–31.1) vs. 2.81 (1.86–3.78) months; median PFS (25th–75th percentile) for NP patients vs. P patients, respectively; Figure 1B). 

Several studies proposed the NLR value as a prognostic biomarker for NSCLC patients, but whether the NLR functions as predictive biomarker for ICI monotherapy response remains unclear [45,46]. We, therefore, initially evaluated whether the patient NLR values at baseline (before initiation of the ICI treatment) correlated with a response to ICI monotherapy in our NSCLC patient cohort. Notably, we did not find any significant difference in the baseline NLR values between the NP and P patients (Figure 1C). More recently, the lung immune prognostic index (LIPI) score (which considers the derived NLR alongside the LDH plasma levels) has been suggested as a more promising candidate biomarker than the NLR itself for predicting ICI monotherapy resistance in lung cancer patients [47,48,49]. In line with this guidance, and with the results observed in the study by Adamo A. et al. using a different cohort of NSCLC patients [44], when we stratified our cohort of NP and P patients according to their baseline LIPI scores (ranging from good (0) to intermediate (1) or poor (2)), we found that the NP patients displayed a significantly lower LIPI score than the P patients (Figure 1D). 

These initial findings supported the recent view that deepening the analysis to develop a more effective combination of several parameters, instead of using single parameter, represents a successful strategy for defining more efficient predictive biomarkers. Indeed, obtaining a better understand of the complex patient immune landscape could provide a more effective strategy for predicting the response to ICI therapy.

### 3.2. Patients with Advanced NSCLC Responding to ICI Monotherapy Displayed Significantly Lower Baseline Absolute Counts of Peripheral Slan^+^-Monocytes, pDCs and NK Cells Than Non-Responding Patients at Baseline

**Figure 2:**
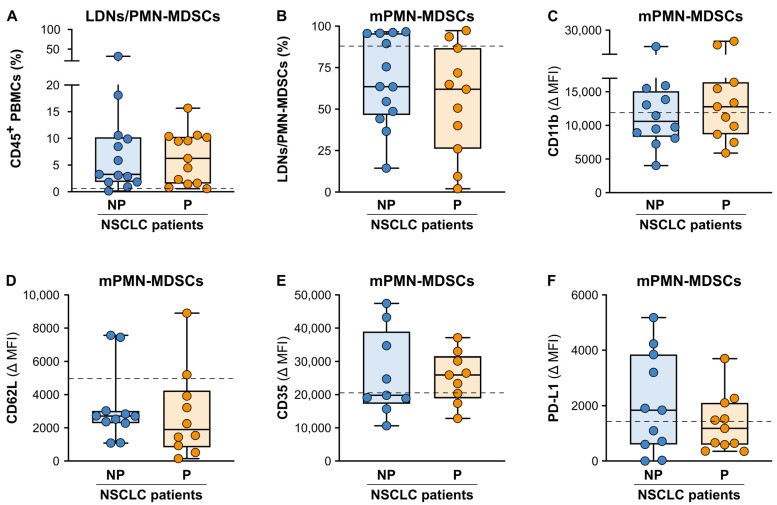
Baseline frequencies and maturation/activation status of the circulating neutrophils and LDNs/PMN-MDSCs from the NP and P advanced NSCLC patients undergoing ICI monotherapy. (**A**) Box plots showing the baseline frequency of the CD66b^+^ LDNs/PMN-MDSCs (within total CD45^+^ PBMCs) in the NP (n = 13) or P (n = 12) NSCLC patients. The median frequency (25th–75th percentile) is reported. The median frequency of the CD66b^+^ LDNs/PMN-MDSCs in the reference HDs is reported as a dashed line. (**B**) Box plots showing the baseline frequency of the mature CD66b^+^CD10^+^ LDNs/PMN-MDSCs (mLDNs/mPMN-MDSCs) (within the total CD66b^+^ LDNs/PMN-MDSCs) in the NP (n = 13) or P (n = 12) NSCLC patients. The median frequency (25th–75th percentile) is reported. Each symbol stands for a single NSCLC patient sample. The median frequency of mature CD66b^+^CD10^+^ LDNs (mLDNs) (within the total CD66b^+^ LDNs) in the reference HDs is reported as a dashed line. (**C**–**F**) The expression levels of CD11b (**C**), CD62L (**D**), CD35 (**E**) and PD-L1 (**F**) were evaluated using flow cytometry on the mature CD66b^+^CD10^+^ LDNs/PMN-MDSCs (mLDNs/mPMN-MDSCs) in the NP (n = 11) or P (n = 10–11) NSCLC patients. Each symbol stands for a single NSCLC patient sample. The median Δ MFI value (25th–75th percentile) for each molecule, calculated as described in the Materials and Methods, is reported. Each symbol stands for a single NSCLC patient sample. (**B**–**F**) The median Δ MFI value of each parameter for reference mature CD66b^+^CD10^+^ LDNs (mLDNs) from the HDs is reported as a dashed line.

To determine the potential blood cell-based biomarkers that could predict the patient clinical response to ICI monotherapy in our cohort of advanced NSCLC patients, we focused our analysis on the evaluation of the baseline frequency/number and activation status of the different populations of innate immune cells, specifically the neutrophils and polymorphonuclear MDSCs (PMN-MDSCs), monocytes and monocytic MDSCs (mo-MDSCs), DCs and NK cells. The PD-L1 (CD274) expressions of the different myeloid cell populations were also evaluated in view of its relevant role in the PD-L1/PD-1 pathway targeted by the ICI monotherapy treatment. Concerning neutrophils, we found no significant differences in the absolute neutrophil counts between our NP and P patient cohorts at baseline, although both the NP and P patients clearly displayed higher neutrophil counts than healthy donors (HDs) (5125 (3528–47,885) and 5230 (3208–7068) vs. 3140 (2400–4530); median neutrophil count/mL (25th–75th percentile) in the NP patients and P patients vs. HDs, respectively). Next, we carefully analyzed the frequency and the phenotype of CD66b^+^ low-density neutrophils (LDNs), known as PMN-MDSCs in cancer patients [40,50,51], according to the gating strategies reported in Figure S1. As shown in Figure 2A, the median frequency of the PMN-MDSCs in both the NP and P patients at baseline was found to be approx. eight times higher than the median frequency of the CD66b^+^ LDNs in HDs (reported as dashed line in Figure 2A). However, no significant differences between the NP and P patients were observed (3.8 (1.8−10.4) and 7.8 (1.5−10.3) vs. 0.6 (0.3−0.8); median of the LDN/PMN-MDSC frequency (25th–75th percentile) in the NP patients and P patients vs. HDs, respectively). Similarly, the frequency of the mature CD66b^+^CD10^+^ LDNs/PMN-MDSCs (Figure S1) [43] within the total CD66b^+^LDNs/PMN-MDSCs (Figure 2B), as well as the expression levels of several activation markers (CD11b, CD62L, CD35; Figure 2C–E) and of PD-L1 (Figure 2F) on the mature CD66b^+^CD10^+^ LDNs/PMN-MDSCs were not different between the NP and P patients. In addition, no significant differences between the expression levels of the same activation markers, as well as of PD-L1, were found when we compared the autologous normal-density neutrophils (NDNs; Figure S1) [52] from the same patient groups and the NDNs from HDs. 

**Figure 3:**
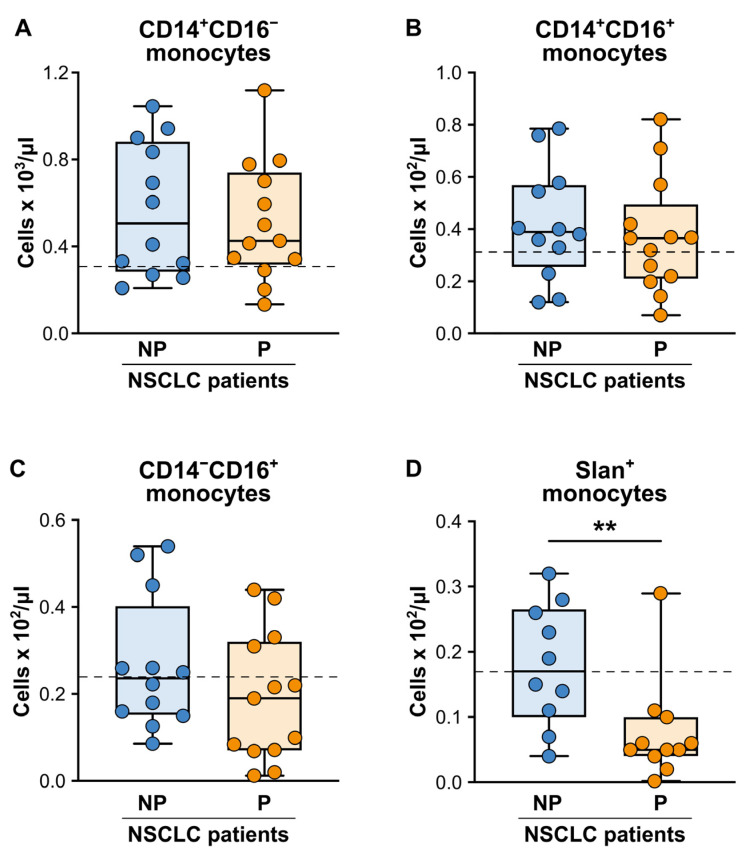
Baseline cell counts of the circulating monocyte subpopulations from the NP and P NSCLC patients undergoing ICI monotherapy. (**A**–**D**) Box plots showing the baseline cell counts of the CD14^+^CD16^−^ classical (**A**), CD14^+^CD16^−^ intermediate (**B**), CD14^low^CD16^+^ non-classical (**C**) slan^+^-monocytes (**D**) in the NP (n = 10–12) or P (n = 11–13) NSCLC patients. The median cell count/mL (25th–75th percentile) for each monocyte population is reported. Each symbol stands for a single NSCLC patient sample. The median cell count/mL of each monocyte population for the reference HDs is reported as a dashed line. ** *p* < 0.01; Mann–Whitney test.

Next, we focused on monocytes, and we found that the NP patients displayed significantly higher total monocyte and CD14^+^CD16^-^ classical monocyte counts (determined according to the gating strategy reported in Figure S2, panel IV) as compared to HDs (820 (495–1225) vs. 500 (407–622)—*p* < 0.01 and 604 (297–921) vs. 304 (189–460)—*p* < 0.05; median total monocyte and CD14^+^CD16^-^ classical monocyte counts/mL (25th–75th percentile) in the NP patients vs. HDs, respectively). However, neither the baseline absolute numbers of the total monocytes (820 (495–1225) vs. 560 (410–892); median total monocyte count/mL (25th–75th percentile) in the NP vs. P patients, respectively), nor the baseline absolute numbers of the CD14^+^CD16^−^ classical (Figure 3A), CD14^+^CD16^−^ intermediate (Figure 3B) or CD14^low^CD16^+^ non-classical (Figure 3C) monocytes (Figure S2, panel IV) were significantly different in the NP patients as compared to the P patients. Despite these initial observations, by performing a more careful evaluation of the additional monocyte subpopulations, we found that the baseline absolute number of slan (MDC8)^+^–monocytes, a subpopulation of proinflammatory monocytes present within CD14^low^CD16^+^ non-classical monocytes [53] (Figure S2, panel V), was strongly reduced in the P patients as compared to the NP patients (Figure 3D). 

**Figure 4:**
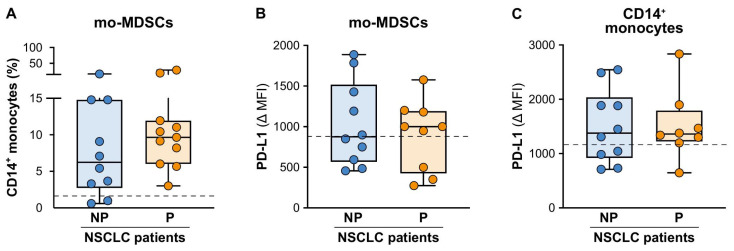
Baseline frequencies and PD-L1 expressions of the circulating mo-MDSCs from the NP and P NSCLC patients undergoing ICI monotherapy. (**A**) Box plots showing the baseline frequency of the circulating CD14^+^HLA-DR^low^ mo-MDSCs (within the total CD14^+^ monocytes) in the NP (n = 11) or P (n = 11) NSCLC patients. The median frequency (25th–75th percentile) is reported. (**B**,**C**) The PD-L1 expression levels were evaluated using flow cytometry on the circulating CD14^+^HLA-DR^low^ mo-MDSCs (**B**) or the total CD14^+^ monocytes (**C**) from the NP (n = 9–10) or P (n = 8–9) NSCLC patients. The median PD-L1 Δ MFI value (25th–75th percentile), calculated as described in the Materials and Methods, for each monocyte population is reported. Each symbol stands for a single NSCLC patient sample. The median frequency of the CD14^+^HLA-DR^low^ mo-MDSCs and the PD-L1 Δ MFI value for the CD14^+^HLA-DR^low^ mo-MDSCs or the total CD14^+^ monocytes for the reference HDs is reported as a dashed line.

On the contrary, and in line with what was observed in a previous study by Adamo A. et al. [44], we did not find significant differences in the baseline frequencies of the CD14^+^HLA-DR^low^ mo-MDSCs (Figure S2, panel VI) between the NP and P patients (Figure 4A), although both patient groups clearly displayed an enhanced frequency of the mo-MDSCs as compared to the HDs (dashed line in Figure 4A; 7.1 (3.3–14.8) and 10.4 (8.2–18.3) vs. 1.5 (0.4−3.3); median mo-MDSC frequencies (25th–75th percentile) in the NP patients and P patients vs. HDs, respectively). Finally, the levels of the PD-L1 expression were not significantly different between the NP and P patients for either the mo-MDSCs (Figure 4B) or CD14^+^ monocytes (Figure 4C). 

**Figure 5:**
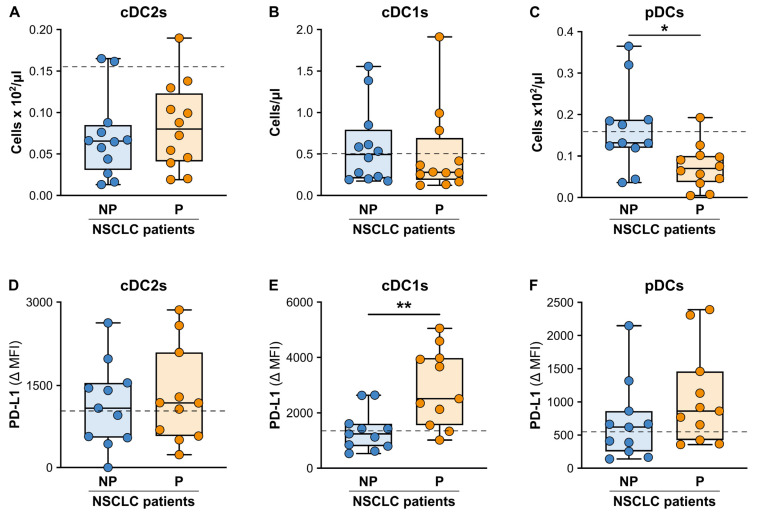
Baseline cell counts and PD-L1 expressions of the circulating DC subpopulations from the NP and P NSCLC patients undergoing ICI monotherapy. (**A**–**C**) Box plots showing the baseline cell counts of the CD141^+^ cDC2s (**A**), CD1c^+^ cDC1s (**B**) and CD303^+^ pDCs (**C**) in the NP (n = 11–12) or P (n = 11–12) NSCLC patients. The median cell count/mL (25th–75th percentile) for each DC population is reported. (**D**–**F**) The PD-L1 expression levels were evaluated using flow cytometry on the circulating CD141^+^ cDC2s (**D**), CD1c^+^ cDC1s (**E**) and CD303^+^ pDCs (**F**) in the NP (n = 11) or P (n = 11) NSCLC patients. The median PD-L1 Δ MFI value (25th–75th percentile), calculated as described in the Materials and Methods, for each DC population is reported. Each symbol stands for a single NSCLC patient sample. The median cell count and PD-L1 Δ MFI value for each DC population for the reference HDs is reported as a dashed line. * *p* < 0.05; ** *p* < 0.01; Mann–Whitney test.

As far as DCs, both the NP and P patients displayed significantly reduced baseline absolute numbers for the total DCs as compared to HDs (20 (14.5–28.2) vs. 36.9 (26.6–56.4)—*p* < 0.05 and 15.3 (8.1–22.4) vs. 36.9 (26.6–56.4)—*p* < 0.05; median total DC count/mL (25th–75th percentile) in the NP patients vs. HDs and P patients vs. HDs, respectively), where CD1c^+^ type 2 conventional DCs (cDC2s; Figure S3, panel IV) were the most strongly reduced DC subtype in all the patients (Figure 5A–C). Although no significant differences in the baseline absolute numbers of the total DCs between the NP and P patients were observed, the baseline absolute count of the CD303^+^ plasmacytoid DCs (pDCs) were significantly reduced in the P patients as compared to the NP patients (Figure 5C) upon a more careful analysis of the DC subtypes (according to the gating strategies reported in Figure S3, panels IV and V). By contrast, no significant differences were present in the baseline absolute counts of the cDC2s (Figure 5A) or of CD141^+^ type 1 cDCs (cDC1s) (Figure 5B). Finally, we found a significantly enhanced expression of PD-L1 on the cDC1s, but not on the cDC2s or pDCs (Figure 5D–F) in the P patients as compared to the NP patients. 

However, no other significant differences on the expression levels of the other activation markers, such as HLD-DR or CD303, were observed on the different DC populations (Figure S4).

**Figure 6:**
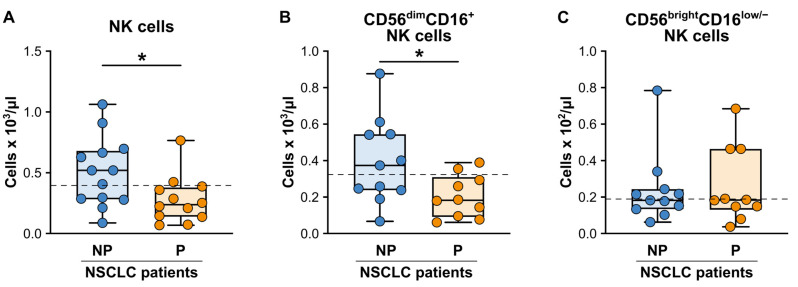
Baseline cell counts of the circulating NK cell subpopulations from the NP and P NSCLC patients undergoing ICI monotherapy. (**A**–**C**) Box plots showing the baseline cell counts of the total (**A**), CD56^dim^CD16^+^ (**B**), CD56^bright^CD16^−^ and (**C**) NK cells in the NP (n = 11–13) or P (n = 10–11) NSCLC patients. The median cell count/mL (25th–75th percentile) for each NK cell population is reported. Each symbol stands for a single NSCLC patient sample. The median cell count/mL of each NK cell population for the reference HDs is reported as a dashed line. * *p* < 0.05; Mann–Whitney test.

Lastly, in line with what was observed in the study by Adamo A. et al. using a different cohort of NSCLC patients [44], the baseline absolute number of the total NK cells was significantly reduced in the P patients as compared to the NP patients (Figure 6A). A more detailed analysis of the two main NK cell subpopulations (analyzed according to the gating strategies reported in Figure S5, panel III), revealed that only the cytotoxic CD56^dim^CD16^+^ NK cell subset was significantly reduced in the P patients as compared to the NP patients (Figure 6B), while the regulatory/cytokine secreting CD56^bright^CD16^-^ NK cell subset was not affected (Figure 6C). 

In summary, these results suggest that, at least in our cohort of advanced NSCLC patients, elevated baseline absolute cell counts of slan^+^-monocytes, pDCs, and NK cells (including the CD56^dim^CD16^+^ NK cell subset), as well as low expression levels of PD-L1 on cDC1s, may function as predictive biomarkers for a positive response to ICI monotherapy. On the other hand, no difference in the baseline absolute number/frequency/activation status of the neutrophils/PMN-MDSCs or the other monocyte/mo-MDSC or DC subsets were found in our cohort of NSCLC patients undergoing ICI monotherapy. 

### 3.3. The Baseline LIPI Score and Absolute Cell Counts of Slan^+^-Monocytes, pDCs, and NK Cells Predicted Clinical Benefit in Advanced NSCLC Patients Treated with ICI Monotherapy

Next, we evaluated whether some of the predictive variables with a significant difference between the NP and P patients at T0, specifically the LIPI score and the cell counts of the slan^+^-monocytes, pDCs, and NK cells, were also correlated with the patients PFS and could, therefore, predict a durable clinical benefit. 

For this purpose, the receiver operating characteristic (ROC) was used to define the optimal baseline cut-off value of the continuous variable (slan^+^-monocyte, pDC, and NK cell baseline cell count) to predict the ICI monotherapy response in our NSCLC patient cohort: slan^+^-monocyte cell counts (0.07 cells × 10^2^/μL; Figure S6A), pDC counts (0.11 cells × 10^2^/μL; Figure S6B), and NK cell counts (0.27 cells × 10^3^/μL; Figure S6C). The prediction performance for each parameter score is shown in Table 2.

**Figure 7:**
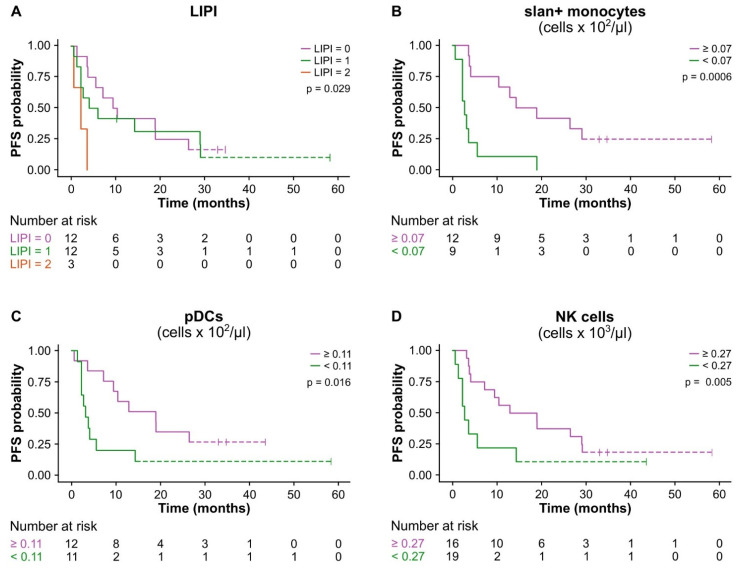
Kaplan–Meier curves displaying the estimated survival probability of NSCLC patients based on the selected blood-based biomarkers. (**A**–**D**) Kaplan–Meier survival curves stratified by the LIPI score (*p* = 0.029, (**A**)), by the optimal cut-off value of the baseline absolute cell counts of the slan^+^-monocytes (0.07 cells × 10^2^/μL; *p* = 0.0006, (**B**)), pDCs (0.11 cells × 10^2^/μL; *p* = 0.016, (**C**)) and NK cells (0.27 cells × 10^3^/μL; *p* 0 0.005, (**D**)) in advanced NSCLC patients treated with ICI monotherapy based on PFS. The censored values are denoted with a tick mark.

Finally, the data from the univariate prognostic factor analysis (Kaplan–Meier curves, Figure 7; Cox regression, Table 3) illustrated that the patients with a baseline LIPI score = 2 (*p* = 0.029, A), slan^+^-monocyte cell counts < 0.07 cells × 10^2^/μL (*p* = 0.0006, Figure 7B), pDC counts < 0.11 cells × 10^2^/μL (*p* = 0.016, Figure 7C), and NK cell counts < 0.27 cells × 10^3^/μL (*p* = 0.005, Figure 7D) displayed a significantly lower PFS.

Overall, these findings showed that a high baseline LIPI score and low cell counts of slan^+^-monocytes, pDCs, and NK cells independently predicted a negative response to therapy and were associated with a clinical benefit in our cohort of NSCLC patients treated with ICI monotherapy.

## 4. Discussion

Over the past decade, ICIs have revolutionized the treatment of NSCLC [8,9]. However, while this therapeutic approach has changed the outcomes for many NSCLC patients, a significant proportion of them do not respond to it [12,16,17]. Thus, identifying predictive biomarkers for the selection of patients who are most likely to respond to ICI treatments would greatly increase the efficacy of ICIs in NSCLC patients, avoid undesirable immunotoxicities, promote the development of precision medicine and decrease treatment costs. 

In this context, significant efforts have been made to identify potential blood-based biomarkers that, compared to tumor microenvironment-related biomarkers, would be non-invasive and relatively cheap [26,27,28]. However, to date, the predictive role of different blood-based biomarkers remains unclear, mostly due to the lack of standard detection/analysis methods and to the fact that most studies have focused on the evaluation of single, or of very few, blood-based biomarkers within the same patient cohort. 

In this study, we evaluated the predictive value of different blood-based biomarkers in the same cohort of NSCLC patients treated with ICI monotherapy. We specifically focused our analysis on the more established blood-based biomarkers that are often utilized in clinical practice, such as the NLR and the LIPI score, as well as on the innate immune cell-related parameters whose prognostic utility in NSCLC patients treated with ICI monotherapy remains poorly investigated or inconsistent. To the best of our knowledge, such a detailed evaluation of several different blood-based biomarkers in the same cohort of NSCLC patients undergoing ICI monotherapy was previously performed only in one study by Moller M. et al. [39]. However, as discussed below, the parameters analyzed in that study and the results obtained only partially overlap with our study. 

The NLR is a measure of systemic inflammation that appears to correlate with patient survival and displays a prognostic role in localized and advanced NSCLC [54,55]. A predictive role of the baseline NLR with regards to the response to ICI monotherapy in NSCLC patients has also been repeatedly proposed [56,57,58,59,60], although it remains controversial [45,46]. In our cohort of NSCLC patients, we did not observe significant differences in the baseline NLR values between the P and NP patients, and we were not able to define an NLR cut-off value that could significantly correlate with patient PFS or a response to therapy. Similar to our study, Moller et al. did not find significant differences in the baseline NLR value in their cohort of NSCLC patients, and they defined an NLR cut-off value of ≥5.2 that correlated with patient PFS; however, it was unsatisfactory as single variable for predicting a response to therapy [39]. It is important to remark that although the pretreatment NLR was often associated with better outcomes in the NSCLC patients undergoing immunotherapy, there was a lack of a uniform cut-off value among the studies [45,46,61]. Furthermore, it is now well documented that the utilization of composite blood-based baseline biomarkers, generated by combining the NLR with other variables that independently correlate with clinical outcomes, provides more precision in predicting a response to ICI therapy than the utilization of the NLR alone [26,62,63]. We, therefore, considered the LIPI score, consisting of the derived NLR and LDH, as a potential predictive biomarker as it currently represents the most studied score with a validated prognostic value in over five thousand advanced NSCLC candidates for ICI therapy [47,48,49]. Interestingly, we found that the baseline LIPI score was significantly higher in the P than NP patients. However, since only a baseline LIPI score of two appeared to be significantly correlated with patient PFS (at least in our NSCLC patient cohort), the predicting value of the LIPI score as a potential blood-based biomarker for ICI monotherapy in NSCLC patients needs to be further evaluated [64]. 

Concerning the blood cell-based biomarkers that were analyzed, we focused mostly on various types of innate immune cell subpopulations, including the neutrophil/PMN-MDSC, monocyte/mo-MDSC, DC and NK cell populations. We observed that the baseline absolute cell counts of the slan^+^-monocytes, pDCs and total NK cells (including the CD56^dim^CD16^+^ NK cell subsets) were significantly lower in the P patients than in the NP patients. Consistently, when we evaluated the predicting value of the different parameters tested based on the patient PFS, we were also able to confirm that, at least in our cohort of NSCLC patients treated with ICI monotherapy, the baseline cut-off values of the slan^+^-monocyte cell count < 0.07 (cells × 10^2^/mL), pDC count < 0.11 (cells × 10^2^/mL) and total NK cell count < 0.27 (cells × 10^3^/^mL^) functioned as independent variables for predicting a negative response to therapy and were correlated with patient PFS. It is important to remark, however, that although our study provided evidence that the pDC, slan^+^-monocyte and NK cell numbers may function as predictive biomarkers of ICI monotherapy in NSCLC patients, a rigorous analytical and clinical validation across a large patient cohort is needed to define the optimal cut-off values that could potentially be used in clinical practice. Furthermore, we found that the baseline expression levels of PD-L1 on the cDC1s, but not on the other analyzed myeloid cell subsets, were significantly increased in the P patients as compared to the NP patients. To the best of our knowledge, the PD-L1 expression on various myeloid cell types was never investigated before as a potential biomarker of ICI monotherapy response. Although the expression levels of specific molecules may not provide accurate cut-off values that could be utilized to predict a patient’s response to therapy (due to the technical variability of the specific instruments/antibodies that are utilized), our findings suggest that monitoring not only the numbers/frequencies of DCs and/or other myeloid cell types but also their activation status and/or potential polarization towards tolerogenic/immunosuppressive phenotypes may provide useful information on the overall patient immune status and the eventual selection of biomarkers for predicting NSCLC patient responses to ICI monotherapy. 

Unlike our study, Moller et al. [39] found that the baseline frequencies, not only for pDCs, but also for cDC1s and cDC2s, were significantly reduced in the P patients as compared to the NP patients. Additionally, Moller et al. [39] showed that the baseline total neutrophil and leukocyte counts were significantly increased in the P patients as compared to the NP patients, while the baseline NK cell counts were not found to be significantly different and slan^+^-monocytes were not investigated. Interestingly, the number/frequency of slan^+^-monocytes were instead proposed by the same research group for correlating a response to treatment in NSCLC [65] and small cell lung cancer (SCLC) [66] patients undergoing ICI therapy combined with chemotherapy. It is important to remark that, similar to their findings on the NLR, Moller et al. showed that the NSCLC patients with a frequency of mo-MDSCs ≥ 11% and/or total DCs % ≤ 0.4 had a significantly lower PFS. However, the cut-off values of these variables were nonetheless unsatisfactory as single variable for predicting a response to therapy [39].

Since both our study and the study by Moller et al. were performed on small cohorts of NSCLC patients and displayed some differences in the types of parameters tested, it is not surprising that the two studies displayed some similar findings as well as some discrepancies. In this context, it is important to remark that the literature was very controversial regarding the potential predictive role of innate cell-based biomarkers for ICI monotherapy in NSCLC patients. For instance, a high frequency of PMN-MDSCs was found to correlate with NSCLC patient responses to ICI monotherapy in some studies [35,36,38], but not to correlate in others [33,34,37]. In this regard, it is also important to remark that due to the lack of commonly accepted PMN-MDSC markers, the PMN-MDSCs were evaluated as Lectin-like oxidized low-density lipoprotein (LDL) receptor-1 (LOX-1)^+^ LDNs [33,34] in some studies, as CD13^+^ LDNs [35] in another, and as the frequency of the total CD66b^+^ LDNs in others, including our study [36,37,38], which further complicated the possibility of comparing the different findings and reaching definitive conclusions. Similarly, the elevated baseline frequencies of mo-MDSCs were found to function as predictive blood-based biomarkers for ICI therapy response in NSCLC patients in some studies [38,40], but not in others [37,39]. The baseline counts of NK cells (or their subsets) were less investigated, but once again described as either predictive [41,42] or not predictive [34,39] blood-based biomarkers in NSCLC patients treated with ICI monotherapy, depending on the study. As far as DCs, to the best of our knowledge, only our study and the study by Moller et al. [39] evaluated the baseline count/frequency of these cells as a potential blood-based biomarker in NSCLC patients treated with ICI monotherapy. Interestingly, both studies suggested that monitoring eventual DC dysfunctions, such as low numbers (especially of pDCs, this study and [39]) or altered phenotypes (as revealed by the enhanced PD-L1 expression on cDC1s in this study) may be indicative of a poor patient immune landscape and a lack of response to ICI monotherapy. Considering, the relevant role that DCs [67], slan^+^-monocytes [68] and NK cells [69] play in sustaining T cell functions and in promoting anti-tumor surveillance, it is indeed plausible that the functional impairment of these cells may reduce the chances of successful outcomes to ICI monotherapy. 

## 5. Conclusions

Overall, the controversies of the studies in the field highlight the lack of uniform standard detection and analysis methods that are fundamental for defining the application of a defined parameter as a potential predictive biomarker for the response to ICI therapy. Additionally, given the complexity of the immune system, a single biomarker may not be suitable to accurately predict the selection of a patient for immunotherapy. As suggested by our study, a detailed evaluation of several blood-based parameters, including the innate immune cell number/activation status, may give a more comprehensive immune profile of individual patients, allowing for a more accurate identification of strong predictive biomarkers. Clearly, however, larger prospective clinical trials are needed to validate whether the innate immune cell number/phenotype may provide novel and promising predictive biomarkers for ICI monotherapy in NSCLC patients. 

## Figures and Tables

**Table 1:** Clinical characteristics of the patients at baseline.

Characteristics	Patients N = 30 (%)	NP N = 15 (%)	P N = 15 (%)
Gender Male Female	20 (66.7) 10 (33.3)	11 (73.3) 4 (26.7)	9 (60.0) 6 (40.0)
Age in years, median (range)	72.5 (43–84)	72 (56–78)	73 (43–84)
ECOG performance status 0 1 2	13 (43.3) 15 (50.0) 2 (6.7)	8 (53.3) 6 (40.0) 1 (6.7)	5 (33.3) 9 (60.0) 1 (6.7)
Smoker Never Former Current	9 (30.0) 12 (40.0) 9 (30.0)	2 (13.3) 6 (40.0) 7 (46.6)	7 (46.7) 6 (40.0) 2 (13.3)
Histology Adenocarcinoma Squamous carcinoma	23 (76.7) 7 (23.3)	11 (73.3) 4 (26.7)	12 (80.0) 3 (20.0)
EGFR status Mutated Wild type	2 (6.7) 28 (93.3)	0 (0.0) 15 (100.0)	2 (13.3) 13 (86.7)
Comorbidities 0 1–3 >3	6 (20.0) 17 (56.7) 7 (23.2)	3 (20.0) 8 (53.3) 4 (26.7)	3 (20.0) 9 (60.0) 3 (20.0)
PD-L1 expression <1% ≥1%–<50% ≥50%	4 (13.3) 9 (30.0) 17 (56.7)	1 (6.7) 3 (20.0) 11 (73.3)	3 (20.0) 6 (40.0) 6 (40.0)
Immunotherapy agent Pembrolizumab Nivolumab Atezolizumab Durvalumab	17 (56.7) 8 (26.7) 3 (10.0) 2 (6.7)	11 (73.3) 1 (6.7) 1 (6.7) 2 (13.3)	6 (40.0) 7 (46.7) 2 (13.3) 0 (0.0)
Line of immunotherapy First Second/third	19 (63.3) 11 (36.7)	13 (86.7) 2 (13.3)	6 (40.0) 9 (60.0)

Abbreviations: N: number; NP: non-progressor; P: progressor; ECOG: Eastern Cooperative Oncology Group; EGFR: epidermal growth factor receptor; PD-L1: programmed death ligand-1.

**Table 2:** Receiver operating characteristic curve analysis for the prediction of therapy response using blood-based parameters.

Prediction Method	Cut-off Point	AUC	95% CI	*p*	Sensitivity (%)	Specificity (%)
Slan^+^-monocytes	0.07 cells × 10^2^/μL	0.8	0.63–1.00	0.01	90	72.73
pDCs	0.11 cells × 10^2^/μL	0.8	0.60–1.00	0.02	81.82	83.33
NK cells	0.27 cells × 10^3^/μL	0.76	0.56–0.95	0.03	84.62	58.33

Abbreviations: AUC indicates the area under the ROC curve; CI, confidence interval.

**Table 3:** Univariate Cox proportional hazard regression model analysis of PFS.

Variable	Value	HR	95% CI	*p*
LIPI	0			
	1	1.26	0.52–3.80	0.60
	2	6.30	1.49–26.70	0.013
Slan^+^-monocytes	≥0.07			
	<0.07	4.99	1.79–13.90	0.002
pDCs	≥0.11			
	<0.11	2.75	1.08–7.0	0.034
NK cells	≥0.27			
	<0.27	2.48	1.01–6.07	0.047

Abbreviations: HR indicates the hazard ratio; CI, confidence interval.

## Data Availability

All the data generated or analyzed during this study are included in this published article and its Supplementary Information files.

## Supplementary Materials

The following supporting information can be downloaded at: https://www.mdpi.com/article/10.3390/cancers15215285/s1, Table S1: Fluorochrome-conjugated antibodies for flow cytometry; Figure S1: Characterization of CD66b^+^LDNs and CD66b^+^NDNs from advanced NSCLC cancer patients and HDs using flow cytometry; Figure S2: Characterization of CD14^+^CD16^−^ classical, CD14^+^CD16^−^ intermediate, and CD14^low^CD16^+^ non-classical monocytes, slan (MDC8)^+^- monocytes and CD14^+^HLA-DR^low^ mo-MDSCs using flow cytometry; Figure S3: Characterization of cDC1s, cDC2s and pDCs using flow cytometry; Figure S4: Baseline HLD-DR and CD303 expression on circulating DC subpopulations from NP and P NSCLC patients undergoing ICI monotherapy; Figure S5: Characterization of the total CD56^+^, CD56^dim^CD16^+^ or CD56^bright^CD16^−^ NK cells using flow cytometry; Figure S6: Receiver operating characteristic (ROC) curves for the prediction of the therapy response based on the baseline value of the predictive markers.
