# Supplementary material for: Plasmacytoid Dendritic Cell, Slan+-Monocyte and Natural Killer Cell Counts Function as Blood Cell-Based Biomarkers for Predicting Responses to Immune Checkpoint Inhibitor Monotherapy in Non-Small Cell Lung Cancer Patients
Source: Cancers (Basel). 2023 Nov 3;15(21):5285. doi: 10.3390/cancers15215285 (PMC10647811; doi:10.3390/cancers15215285)
Supplement: Supplementary file 1 [file cancers-15-05285-s001.zip › cancers-2637015-supplementary.pdf]

cancers

Supplementary Materials

Plasmacytoid Dendritic Cell, Slan$^+$-Monocyte and Natural Killer Cell Counts Function as Blood Cell-Based Biomarkers for Predicting Responses to Immune Checkpoint Inhibitor Monotherapy in Non-Small Cell Lung Cancer Patients

Francesca Pettinella [1], Chiara Lattanzi [1], Marta Donini [1], Elena Caveggion [1], Olivia Marini [1], Giulia Iannoto [1], Sara Costa [1], Elena Zenaro [1], Tiago Moderno Fortunato [1], Sara Gasperini [1], Matteo Giani [1], Lorenzo Belluomini [2], Marco Sposito [2], Jessica Insolda [2], Ilaria Mariangela Scaglione [2], Michele Milella [2], Annalisa Adamo [3], Ornella Poffe [3], Vincenzo Bronte [4], Stefano Dusi [1], Marco A. Cassatella [1], Stefano Ugel [3], Sara Pilotto [2] and Patrizia Scapini [1,*]

[1] General Pathology Section, Department of Medicine, University of Verona, 37134 Verona, Italy
[2] Section of Innovation Biomedicine—Oncology Area, Department of Engineering for Innovation Medicine (DIMI), University of Verona and University and Hospital Trust (AOUI) of Verona, 37134 Verona, Italy
[3] Immunology Section, Department of Medicine, University and Hospital Trust (AOUI) of Verona, 37134 Verona, Italy
[4] Veneto Institute of Oncology—Istituto di Ricovero e Cura a Carattere Scientifico (IOV-IRCCS), 35128 Padova, Italy
* Correspondence: patrizia.scapini@univr.it; Tel.: +39-045-8027-556; Fax: +39-045-8027-127

Table S1. Fluorochrome-conjugated antibodies for flow cytometry.

Target	Clone	Label	Source	Identifier
CD56	REA196	PE	Miltenyi Biotec	Cat# 130-112-312, RRID: AB_2726090
CD45	HI30	BV510	BioLegend	Cat# 304036, RRID: AB_2561940
	5B1	VioGreen	Miltenyi Biotec	Cat# 130-113-124, RRID: AB_2725952
CD66b	G10F5	FITC	BioLegend	Cat# 305104, RRID: AB_314496
	G10F5	APC	BioLegend	Cat# 305118, RRID: AB_2566607
	G10F5	PerCP-Cy5.5	BioLegend	Cat# 305108, RRID: AB_2077855
	6/40c	BV421	BioLegend	Cat# 392916, RRID: AB_2888722
CD11b	ICRF44	PE-Cy7	BioLegend	Cat# 301322, RRID: AB_830644
	ICRF44	APC-Cy7	BD Bioscience	Cat# 557754, RRID: AB_396860
CD16	3G8	APC-Cy7	BioLegend	Cat# 301018, RRID: AB_314218
CD16	3G8	PE-Cy7	BD Bioscience	Cat# 557744, RRID: AB_396850
CD10	HI10a	PE	BioLegend	Cat# 312204, RRID: AB_314915
	HI10a	APC	BioLegend	Cat# 312210, RRID: AB_314921
CD62L	145/15	APC	Miltenyi Biotec	Cat# 130-113-617, RRID: AB_2733392
CD14	TÜK4	VioBlue	Miltenyi Biotec	Cat# 130-113-152, RRID: AB_2725980
CD11c	3.9	PE	eBioscience	Cat# 12-0116-42, RRID: AB_10597432
CD1c	REA694	Vio Bright FITC	Miltenyi Biotec	Cat# 130-110-598, RRID: AB_2656035
CD303	AC144	FITC	Miltenyi Biotec	Cat# 130-113-192, RRID: AB_2726017
CD141	REA674	PE-Vio770	Miltenyi Biotec	Cat# 130-113-663, RRID: AB_2751169
CD3	REA613	PE-Vio770	Miltenyi Biotec	Cat# 130-113-140, RRID: AB_2725968
CD19	HIB19	PE-Cy7	BioLegend	Cat# 302216, RRID: AB_314246
M-DC8	DD-1	FITC	Miltenyi Biotec	Cat# 130-117-371, RRID: AB_2733608
CD274 / PD-L1	MIH1	BV421	BD Bioscience	Cat# 563738, RRID: AB_2738396
HLA-DR	L243	APC	BioLegend	Cat# 307610, RRID: AB_314688
	L243	APC-Cy7	BioLegend	Cat# 307618, RRID: AB_493586

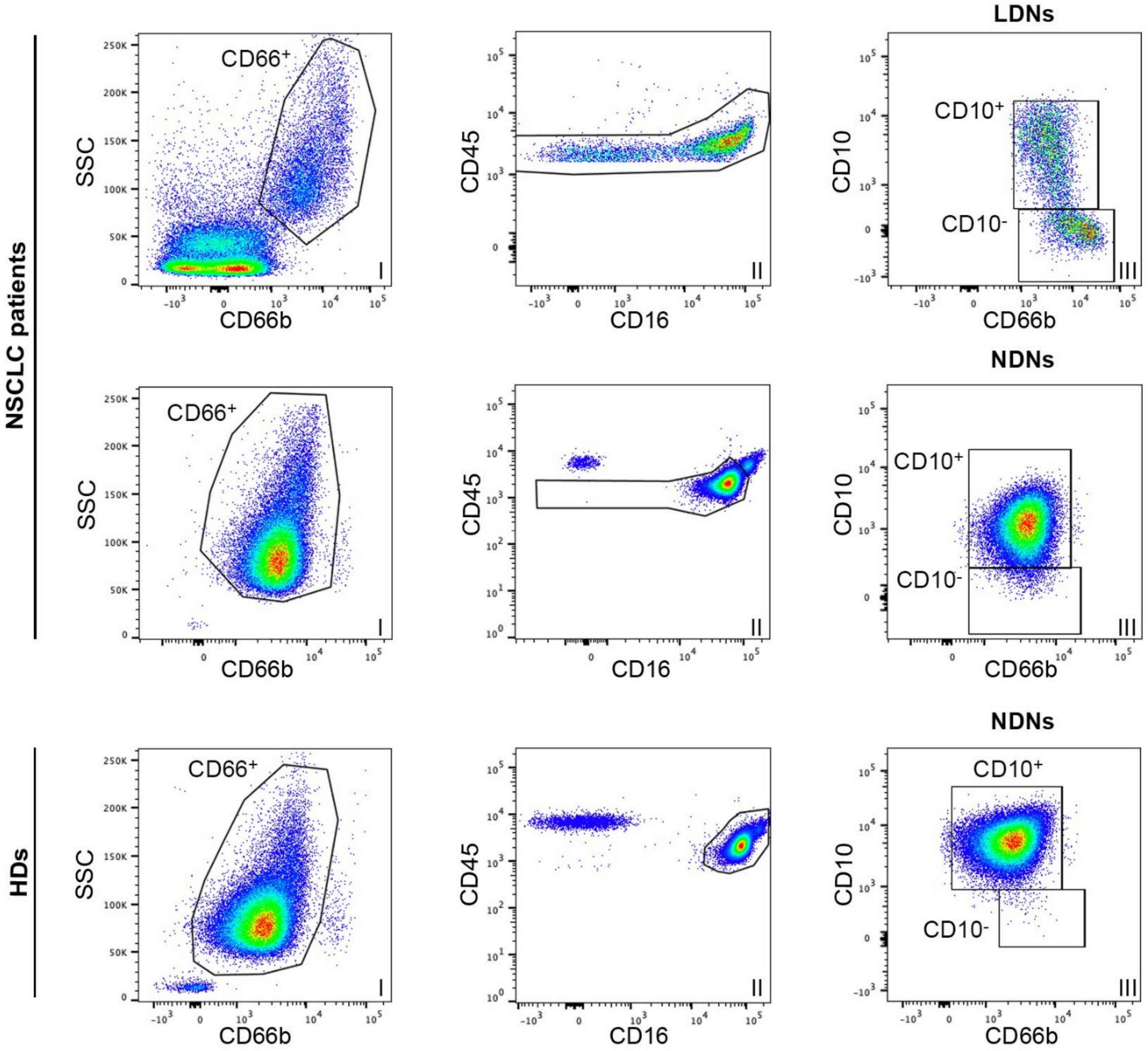

Figure S1. Identification of CD66b⁺LDNs and CD66b⁺NDNs from advanced NSCLC patients and HDs by flow cytometry. After gating on singlets, excluding debris and gating on CD45$^+$Sytox$^-$ cells (not shown), CD66b$^+$CD10$^{+/-}$CD16^{high} LDNs/NDNs from NSCLC patients (top and middle panels) and CD66b$^+$CD10$^{+/-}$CD16^{high} NDNs from HDs (bottom panels) were identified by gating on total CD66b$^+$ granulocytes (I), excluding CD45^{high}CD16$^-$ eosinophils (II) and finally gating on CD10$^{+/-}$ LDNs/NDNs (III).

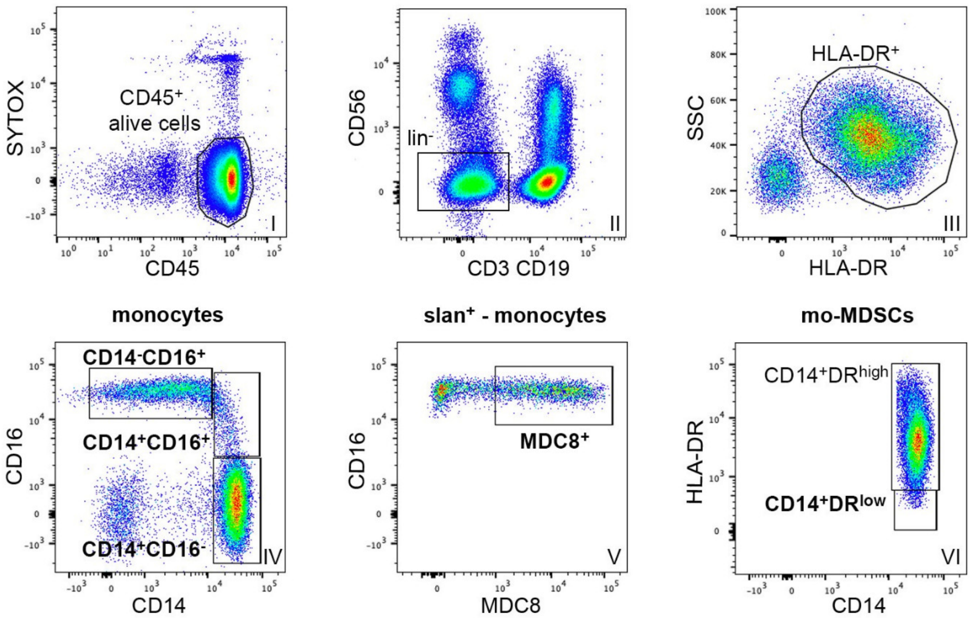

Figure S2. Characterization of CD14^{+}CD16^{-} classical, CD14^{+}CD16^{-} intermediate, and CD14$^{dim/-}$CD16^{+} non-classical monocytes, slan (MDC8)$^{+}$- monocytes and CD14^{+}HLA-DR^{low} mo-MDSCs by flow cytometry. After gating on singlets (not shown), excluding debris (not shown), gating on CD45^{+}Sytox^{-} cells (I), gating on lin^{-} (CD13^{-}CD19^{-}CD56^{-}) cells (II), gating on HLA-DR^{+} cells (III), CD14^{+}CD16^{-} classical, CD14^{+}CD16^{-} intermediate, and CD14$^{dim/-}$CD16^{+} non-classical monocytes (IV), slan (MDC8)$^{+}$-monocytes (V) and CD14^{+}HLA-DR^{low} mo-MDSCs (VI) were identified as reported in the representative plots.

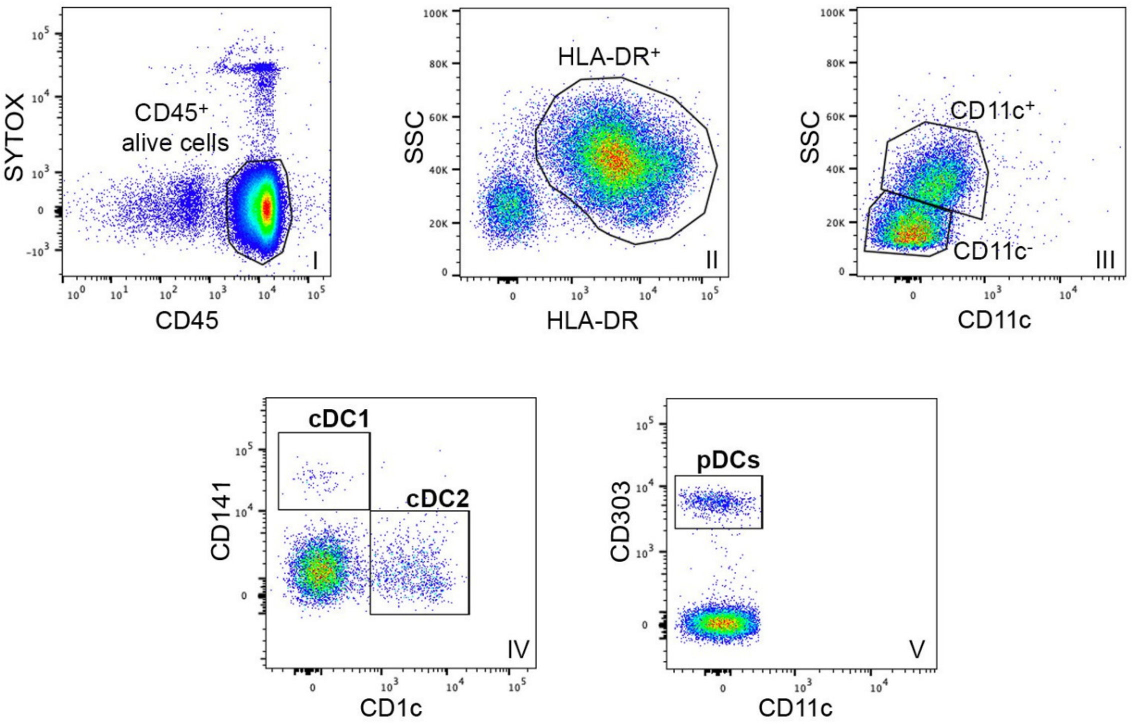

Figure S3. Characterization of cDC1s, cDC2s and pDCs by flow cytometry. After gating on singlets (not shown), excluding debris (not shown), gating on CD45^{+}Sytox^{-} cells (I), gating on HLA-DR^{+} cells (II), cDC1s and cDC2s were identified as CD141^{+} cells (cDC1s, IV) or CD1c^{+} cells (cDC2, IV), respectively, within total CD11c^{high} cells (III), while pDCs were identified as CD303^{+} cells within total CD11c$^{low/-}$ cells (III, V).

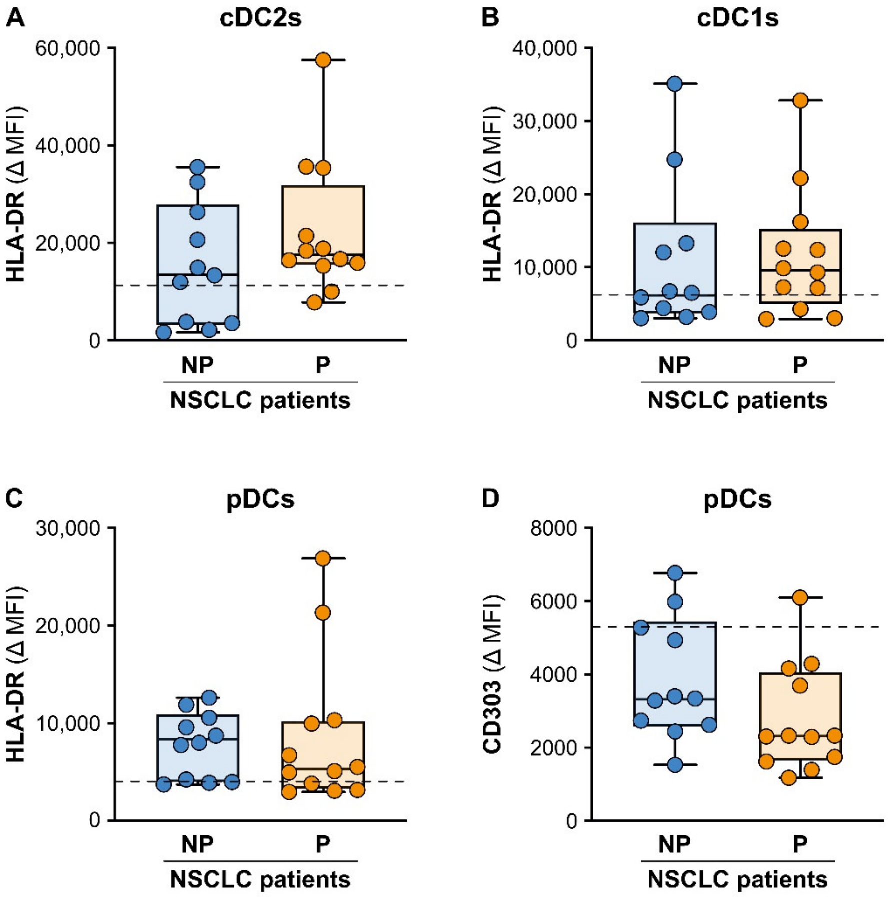

Figure S4. Baseline HLD-DR and CD303 expression on circulating DC subpopulations from NP and P NSCLC patients undergoing ICI monotherapy. HLA-DR (A - C) and CD303 (D) expression levels were evaluated by flow cytometry on circulating CD141$^+$ cDC2s (A), CD1c$^+$ cDC1s (B) and CD303$^+$ pDCs (C, D) in NP (n = 11) or P (n = 12) NSCLC patients. The median HLA-DR (A - C) or CD303 (D) Δ MFI value (25th - 75th percentile), calculated as described in Methods, for each DC population is reported. Each symbol stands for a single NSCLC patient sample. The median HLA-DR (A - C) or CD303 (D) Δ MFI value for each DC population for reference HDs is reported as dashed line.

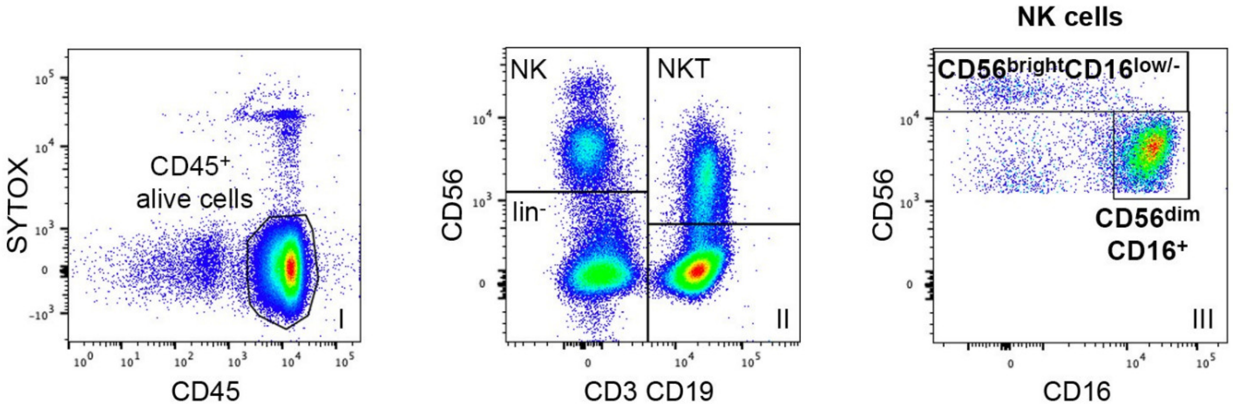

Figure S5. Characterization of total CD56$^+$, CD56^{dim}CD16$^+$ or CD56^{bright}CD16$^-$ NK cells by flow cytometry. After gating on singlets (not shown), excluding debris (not shown), gating on CD45$^+$Sytox$^-$ cells (I), total CD56$^+$ (II), CD56^{dim}CD16$^+$ (III) or CD56^{bright}CD16$^-$ (III) NK cells were identified as reported in the representative plots.

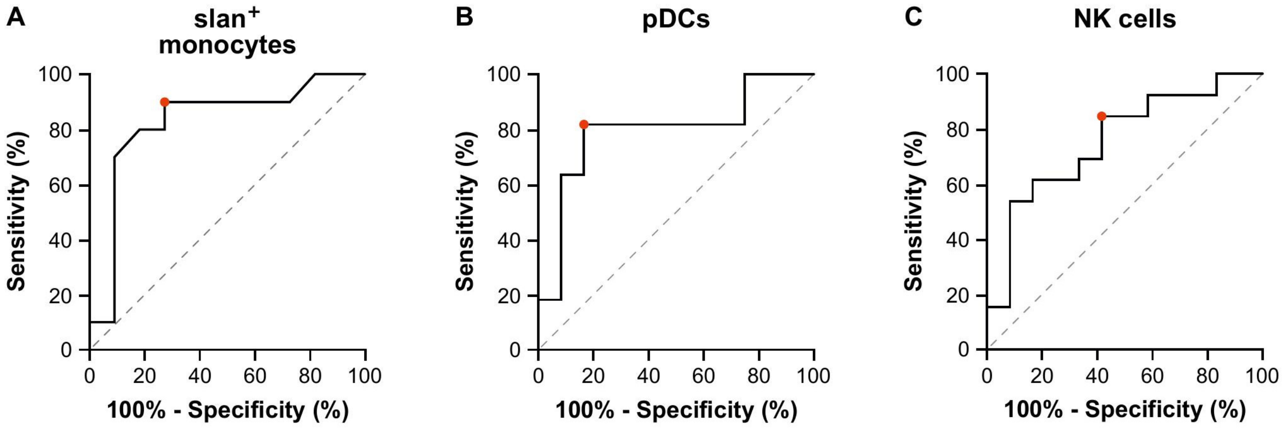

Figure S6. Receiver operating characteristic (ROC) curves to assess therapy responses based on baseline values of predictive biomarkers. (A - C) ROC curves for prediction of the therapy response based on baseline cell counts of slan⁺-monocytes (A), pDCs (B) and total NK cells (C) are shown. The optimal cut-off for each parameter (red dot) is based on trade-off between specificity and sensitivity.
